# Glutathione metabolism in *Cryptocaryon irritans* involved in defense against oxidative stress induced by zinc ions

**DOI:** 10.1186/s13071-022-05390-9

**Published:** 2022-09-07

**Authors:** Zhi-Hong Zhong, Zhi-Cheng Li, Han Li, Qing-Kai Guo, Chen-Xi Wang, Ji-Zhen Cao, An-Xing Li

**Affiliations:** State Key Laboratory of Biocontrol/Guangdong Provincial Key Laboratory of Improved Variety Reproduction in Aquatic Economic Animals and Institute of Aquatic Economic Animals, School of Life Sciences, Guangdong Province, Sun Yat-Sen University, Guangzhou, 510275 People’s Republic of China

**Keywords:** *Cryptocaryon irritans*, Glutathione metabolism, Oxidative stress, Metabolomics, Zinc ions

## Abstract

**Background:**

*Cryptocaryon irritans* is a fatal parasite for marine teleosts and causes severe economic loss for aquaculture. Galvanized materials have shown efficacy in controlling this parasite infestation through the release of zinc ions to induce oxidative stress.

**Methods:**

In this study, the resistance mechanism in *C. irritans* against oxidative stress induced by zinc ions was investigated. Untargeted metabolomics analysis was used to determine metabolic regulation in *C. irritans* in response to zinc ion treatment by the immersion of protomonts in ZnSO_4_ solution at a sublethal dose (20 μmol). Eight differential metabolites were selected to assess the efficacy of defense against zinc ion stimulation in protomonts of *C. irritans*. Furthermore, the mRNA relative levels of glutathione metabolism-associated enzymes were measured in protomonts following treatment with ZnSO_4_ solution at sublethal dose.

**Results:**

The results showed that zinc ion exposure disrupted amino acid metabolism, carbohydrate metabolism, lipid metabolism, and nucleotide metabolism in *C. irritans*. Four antioxidants, namely ascorbate, S-hexyl-glutathione, syringic acid, and ubiquinone-1, were significantly increased in the Zn group (*P* < 0.01), while the glutathione metabolism pathway was enhanced. The encystment rate of *C. irritans* was significantly higher in the ascorbate and methionine treatment (*P* < 0.05) groups. Additionally, at 24 h post-zinc ion exposure, the relative mRNA level of glutathione reductase (*GR*) was increased significantly (*P* < 0.01). On the contrary, the relative mRNA levels of glutathione S-transferase (*GT*) and phospholipid-hydroperoxide glutathione peroxidase (*GP*x) were significantly decreased (*P* < 0.05), thus indicating that the generation of reduced glutathione was enhanced.

**Conclusions:**

These results revealed that glutathione metabolism in *C. irritans* contributes to oxidative stress resistance from zinc ions, and could be a potential drug target for controlling *C. irritans* infection.

**Graphical Abstract:**

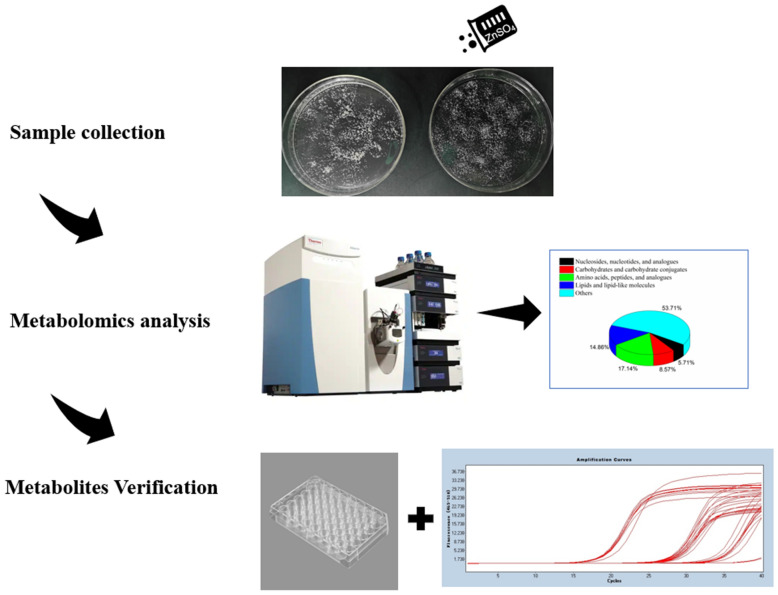

**Supplementary Information:**

The online version contains supplementary material available at 10.1186/s13071-022-05390-9.

## Background

*Cryptocaryon irritans* is an important parasitic ciliated protozoon of numerous marine fish species that causes a fatal disease [1]. The *C. irritans* life-cycle comprises theronts, trophonts, protomonts, and tomonts [2, 3]. Theronts released from tomonts can rapidly invade host skin and gills, where they develop to trophonts. During this process, asphyxiation, osmotic imbalance, and secondary bacterial infections occur in the host fish, contributing to the high mortality [3, 4]. This parasite brings enormous economic loss and is a severe threat to the development of marine aquaculture [1]. Unfortunately, current control strategies have been unable to resolve this problem; therefore, more efficient and sustainable methods are urgently needed.

In our previous study, galvanized materials were found to efficiently control *C. irritans* infestation [5]. The mechanism of galvanized material toxicity was the induction of oxidative stress in *C. irritans* from the released zinc ions [6]. Reactive oxygen species (ROS) content in *C. irritans* increased following zinc ion treatment. The increase in ROS may be because ROS production exceeds the capacity of the antioxidant defense system [6]. This system is vital for *C. irritans* survival following exposure to chemicals. Therefore, the antioxidant defense system could be a target to weaken *C. irritans* resistance to environmental stimulation such as chemicals and host immune clearance.

The antioxidant defense system consists of antioxidant enzymes including superoxide dismutase (SOD) and catalase (CAT), and antioxidant substances such as glutathione and vitamin C [7]. Glutathione is an important antioxidant and an essential cofactor for antioxidant enzymes, providing protection for functional proteins via the redox buffer for sulfhydryl [8, 9]. A disturbance to glutathione homeostasis may lead to oxidative damage and disease, such as neurodegenerative disorders [10]. However, whether glutathione is involved in oxidative resistance in *C. irritans* is not well understood.

Metabolomics is an emerging tool for gaining insights into cellular and physiological responses [11], and has been used to investigate the interactions between metals and organisms [12]. For example, Sinclair et al. used metabolomics to assess the metabolic responses to zinc and boscalid in *Simplisetia aequisetis* [13]. In this study, metabolomic analysis was used to explore the resistance mechanism of *C. irritans* to oxidative stress induced by zinc ions. The role of glutathione metabolism in the defense of *C. irritans* against zinc stress was also determined.

## Methods

### Isolation, propagation, and collection of parasites

The original *C. irritans* strain GD1 was isolated from cultured pompano *Trachinotus ovatus* (500–800 g) and propagated in the laboratory following the methods described by Dan et al. [14]. The protomont stage was used to test the response to zinc ion stress, and protomonts were collected following previously reported methods [6]. Briefly, infected fish were collected and placed in a clean tank before the parasitic *C. irritans* automatically detached from host fish. A number of protomonts were collected at intervals of 1 h and used for testing.

### Metabolome preparation

Protomonts were exposed to sublethal ZnSO_4_ solution (20 μmol; Rahwn, China) for 24 h, then sampled and stored in liquid nitrogen. ZnSO_4_-free seawater served as control. Each group included six replicates (100 mg/replicate). All samples were homogenized by a tissue grinder (Mei Bi, MB-96) at 50 Hz for 60 s. Methanol (0.6 ml) was added, followed by vortexing for 30 s. The mixture was ground again for 60 s at 50 Hz, and then centrifuged at 4 °C for 10 min at 12,000 rpm. The supernatant was filtered through a 0.22-µm membrane to obtain the samples for liquid chromatography–mass spectrometry (LC–MS) and quality control [15, 16].

### High-performance LC–MS analysis

LC–MS (UltiMate 3000 coupled with Q Exactive, Thermo Fisher) was used for sample analysis. Chromatographic separation was undertaken using an Acquity HSS T3 UPLC (ultra-performance LC; inner diameter 150 × 2.1 mm, particle size 1.8 µm, Waters) column maintained at 40 °C. Gradient elution of analytes was carried out with 0.1% formic acid in water (C) and 0.1% formic acid in acetonitrile (D), or 5 mM ammonium formate in water (A) and acetonitrile (B) at a flow rate of 0.25 ml/min. The injection of 2 μl of each sample was done after equilibration, and the autosampler temperature was 8 °C. An increasing linear gradient of solvent B (v/v) was used as follows: 0–1 min, 2% B/D; 1–9 min, 2–50% B/D; 9–12 min, 50–98% B/D; 12–13.5 min, 98% B/D; 13.5–14 min, 98–2% B/D; 14–20 min, 2% D-positive mode (14–17 min, 2% B-negative mode). Electrospray ionization–tandem mass spectrometry (ESI-MS/MS) experiments used a spray voltage of 3.5 kV and −2.5 kV in positive and negative modes, respectively. Sheath gas and auxiliary gas were set at 30 and 10 arbitrary units, respectively. The capillary temperature was 325 °C. The Orbitrap analyzer scanned over a mass range of *m*/*z* 81–1000 for a full scan at a mass resolution of 70,000. Data-dependent acquisition (DDA) MS/MS experiments were performed with a higher-energy C-trap dissociation cell (HCD) scan. The normalized collision energy was 30 eV. Dynamic exclusion was implemented to remove some unnecessary information in the MS/MS spectra [17, 18].

### Data analysis

Peak identification, filtration, and alignment were conducted in the XCMS package in R [19]. After batch normalization of peak intensity, a multivariate analysis was carried out using principal component analysis (PCA), partial least squares discriminant analysis (PLS–DA), and orthogonal projections to latent structures discriminant analysis (OPLS–DA) to identify differences between the Zn group and the control [20]. Metabolites with *P*-values ≤ 0.05 and variable importance in projection (VIP) ≥ 1 in the difference were considered as biomarkers [21]. Differential metabolites were annotated according to METLIN (http://metlin.scripps.edu) and MoNA [MassBank of North America] (https://mona.fiehnlab.ucdavis.edu/). The *Z*-score was calculated to show differences in the metabolites [22]. MetPA [Metabolomic Pathway Analysis], a part of the MetaboAnalyst platform, was used for the Kyoto Encyclopedia of Genes and Genomes (KEGG) pathway analysis of differential metabolites [23].

### Verification of metabolite function

To evaluate the effect of different metabolites on the resistance of *C. irritans* to zinc ion stress, eight differential metabolites were selected based on the significant reduction in their content after zinc ion treatment (mannitol, heptanoic acid, l-methionine, mandelic acid, 5,6-dihydro-5-fluorouracil, nicotinic acid mononucleotide, and pipecolic acid) and reported antioxidant function (ascorbate, all purchased from Rahwn, China). Protomonts were added to a 48-well cell cultivation plate (800 μl seawater, 20 cells/well), followed by 100 μl of differential metabolite solution. After 30 min, 100 μl ZnSO_4_ solution was added. The final concentration of differential metabolites and ZnSO_4_ in the tested solution was 100 μmol/l. The plate was cultivated at 26–28 °C for 7 days, and finally the excystment rate was recorded, as follows: excystment rate = (number of excysted tomonts/total number of protomonts) × 100%.

### qRT-PCR for glutathione metabolism-associated enzymes

To assess the response of glutathione metabolism-associated enzymes to oxidative stress in *C. irritans*, the expression of related genes in *C. irritans* was detected by quantitative real-time polymerase chain reaction (qRT-PCR) after zinc ion treatment. Measured genes were glutamate-cysteine ligase, catalytic subunit (*GC*), glutathione synthase (*GS*), glutathione reductase (*GR*), glutathione S-transferase (*GT*), and phospholipid-hydroperoxide glutathione peroxidase (*GPx*). Protomonts were treated with ZnSO_4_ solution (20 μmol/l) or ZnSO_4_-free seawater (control), and then sampled at 6, 12, and 24 h. Every group was run in triplicate. Samples were immersed in RNAlater stabilization solution (Thermo Fisher) and stored at −80 °C until RNA extraction. Total RNA extraction, reverse transcription, and gene quantification were carried out using the RNeasy Plus Mini Kit (QIAGEN), Evo M-MLV kit (Accurate Biology Co., LTD, China) and SYBR Green Pro Taq HS kit (Accurate Biology Co., LTD, China) respectively. The primers used in the qRT-PCR are listed in Additional file 13: Table S1. EF-1Beta was used as an endogenous control to normalize the gene expression. The relative expression of the target gene was calculated by the 2^−△△Ct^ method.

### Statistical analysis

Differences in metabolite content between the Zn group and control were considered significant at *P* < 0.05 and VIP ≥ 1. Every group was run in triplicate and represented as mean ± standard deviation. Student’s *t*-test was used to determine the difference in excystment rate and relative gene expression between the Zn group and control using Microsoft Office Excel 2019. *P* ≤ 0.05 and *P* ≤ 0.01 were considered to indicate significant and highly significant differences.

## Results

### Metabolic changes in *C. irritans* after zinc ion treatment

**Fig. 1:**
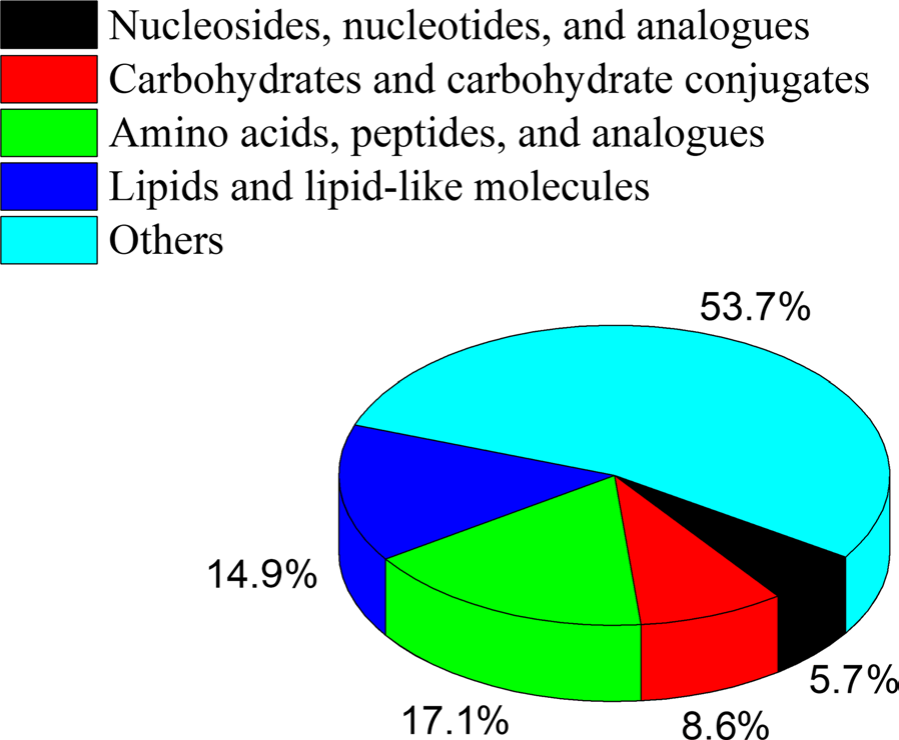
Categories of differential metabolites in *Cryptocaryon irritans* when treated with zinc ions

**Fig. 2:**
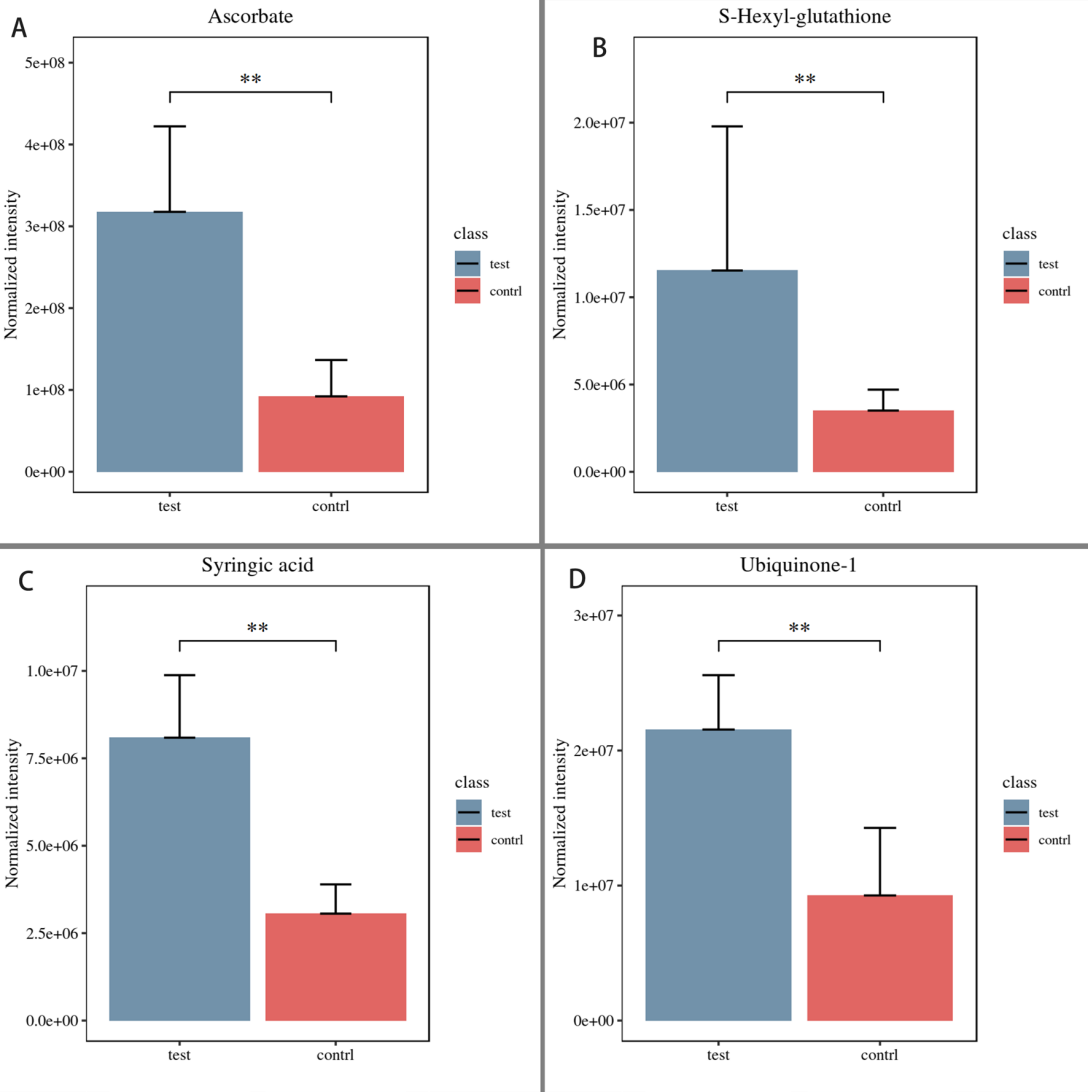
Four antioxidants in the response of *Cryptocaryon irritans* to zinc ion stress*.* The content of all four increased significantly after treatment with zinc ions. **Highly significant in difference (*P* < 0.01)

The response of *C. irritans* metabolism to zinc ion treatment was investigated by metabolomics. A total of 13,311 and 10,091 precursor molecules were identified in positive and negative modes, respectively. Multivariate analysis showed a significant difference between the Zn and control groups, with clear clustering of samples (Additional file 1: Figure S1). A total of 2727 metabolites in the Zn group were significantly different compared with the control: 1847 were upregulated and 880 were downregulated. Among these differential metabolites, nucleosides, nucleotides, and analogs accounted for 5.71%; carbohydrates and carbohydrate conjugates for 8.57%; lipids and lipid-like molecules for 14.86%; and amino acids, peptides, and analogs for 17.14% (Fig. 1). Among the amino acids, peptides, and analogs, the largest increase and decrease were in 5-aminopentanoic acid and beta-tyrosine, respectively (Additional file 2: Figure S2). In the carbohydrates and carbohydrate conjugates, kyotorphin and alpha-d-glucose showed the largest increase and decrease, respectively (Additional file 3: Figure S3). In the lipids and lipid-like molecules and nucleosides, nucleotides and analogs, the metabolites with the greatest increase were glutarate semialdehyde and deoxyuridine, while the metabolites with the greatest decrease were heptanoic acid and nicotinic acid mononucleotide, respectively (Additional file 4: Figure S4, Additional file 5: Fig. S5). Additionally, the content of four antioxidants, namely ascorbate, glutathione, syringic acid, and ubiquinone-1, was higher in the Zn group (Fig. 2, *P* < 0.05).

**Fig. 3:**
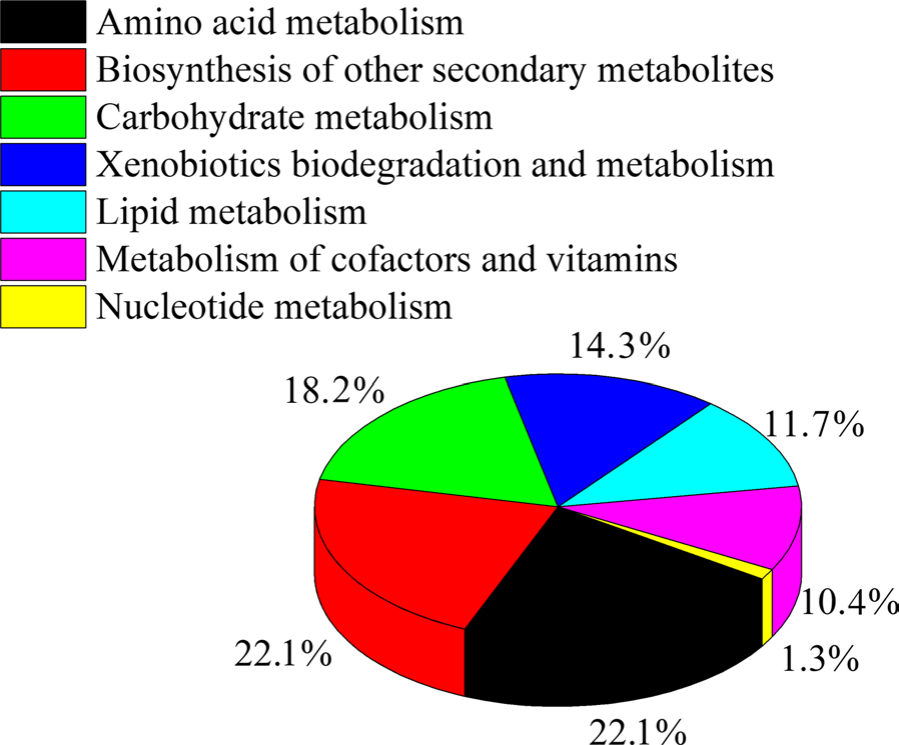
Categories of metabolic pathways in *Cryptocaryon irritans* affected by exposure to zinc ions

Furthermore, according to the KEGG database, differential metabolites were enriched in the following metabolic pathways: amino acid metabolism, biosynthesis of other secondary metabolites, carbohydrate metabolism, xenobiotic biodegradation and metabolism, lipid metabolism, cofactor and vitamin metabolism, and nucleotide metabolism, accounting for 22.08%, 22.08%, 18.18%, 14.29%, 11.69%, 10.39%, and 1.3%, respectively (Fig. 3). In these pathway categories, the most highly affected pathways were valine, leucine, and isoleucine biosynthesis (Additional file 6: Figure S6); tropane, piperidine, and pyridine alkaloid biosynthesis (Additional file 7: Figure S7); beta-alanine metabolism (Additional file 8: Figure S8); aminobenzoate degradation (Additional file 9: Figure S9); linoleic acid metabolism (Additional file 10: Figure S10); pantothenate and coenzyme A (CoA) biosynthesis (Additional file 11: Figure S11); and pyrimidine metabolism (Additional file 12: Figure S12). Furthermore, the glutathione metabolism pathway was enhanced in *C. irritans* exposed to zinc ions (Additional file 6: Figure S6).

### Glutathione metabolism in *C. irritans* resistance to oxidative stress

**Fig. 4:**
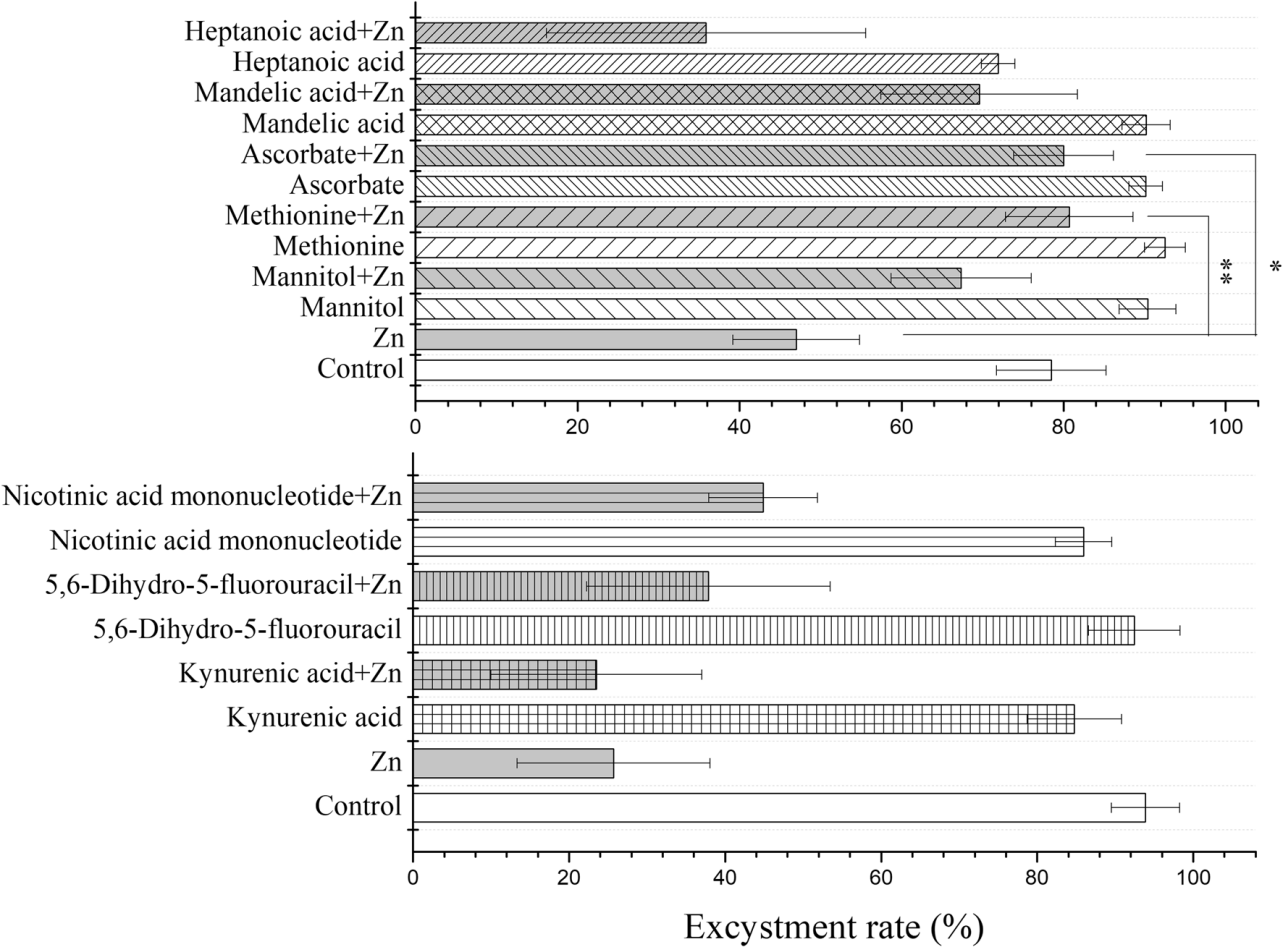
Effect of adding different exogenous metabolites on the excystment rate of *Cryptocaryon irritans*. *Significant differences (*P* < 0.05); **highly significant (*P* < 0.01) differences

**Fig. 5:**
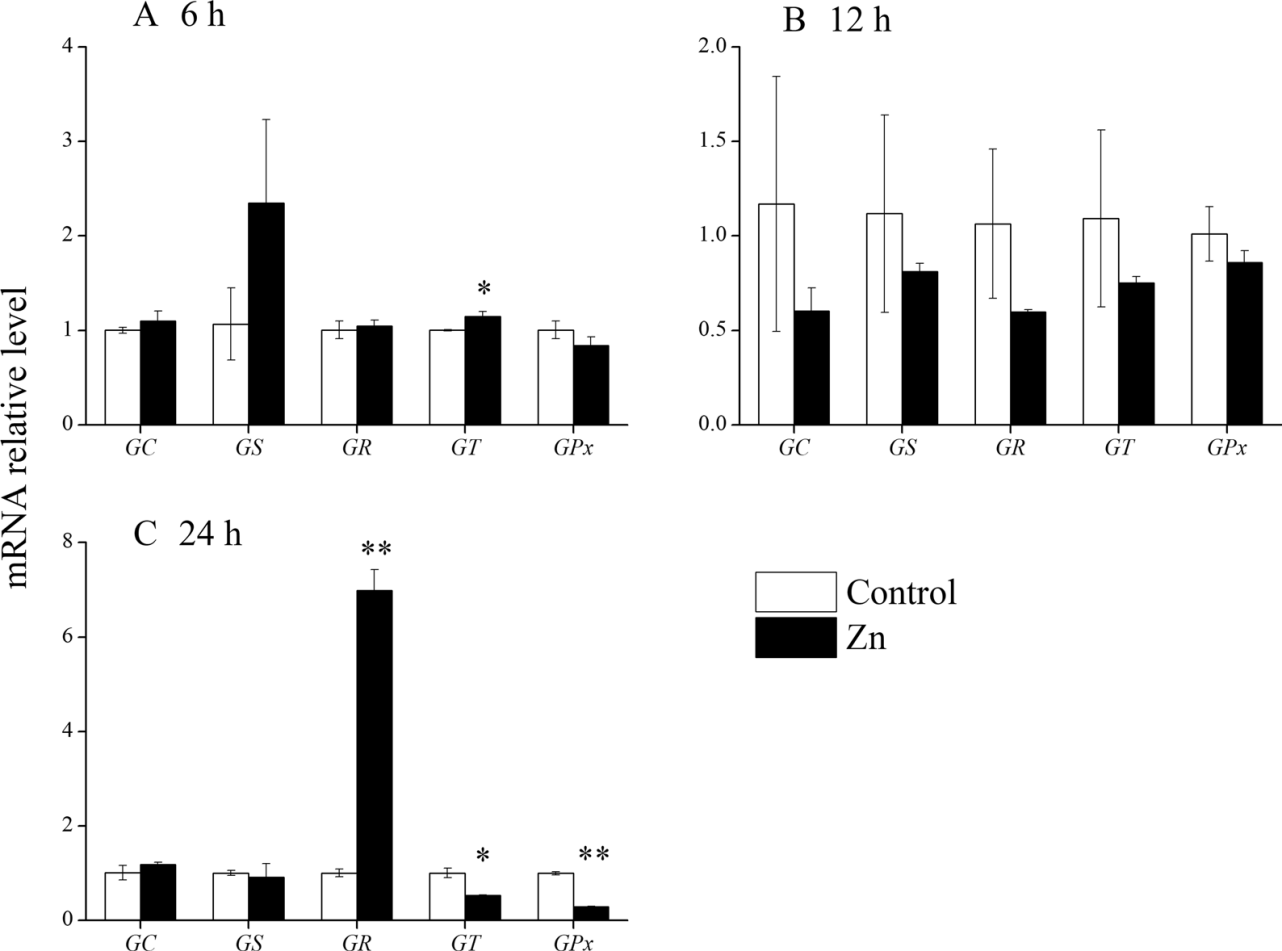
The relative expression of glutathione metabolism-associated genes. *Significant differences (*P* < 0.05); **highly significant differences (*P* < 0.01). *GC* glutamate-cysteine ligase, catalytic subunit; *GS* glutathione synthase; *GR* glutathione reductase; *GT* glutathione S-transferase; *GPx* phospholipid-hydroperoxide glutathione peroxidase

Eight differential metabolites were selected to evaluate their efficacy in assisting *C. irritans* in defense against oxidative stress induced by zinc ions. The excystment rate on the Zn + ascorbate group was lower than that of the Zn group (*P* < 0.05) and the Zn + methionine group (*P* < 0.01, Fig. 4). No significant differences in excystment rates were observed for the other differential metabolites. This indicates that oxidative stress in *C. irritans* was relieved by adding ascorbate and methionine. Additionally, the expression of glutathione metabolism-associated enzymes was measured in *C. irritans* treated with zinc ions after 6, 12, and 24 h. At 6 h post-treatment, only the relative expression of *GT* had increased significantly (*P* < 0.05, Fig. 5). At 12 h post-treatment, no genes were differentially expressed. However, at 24 h post-treatment, a highly significant increase in the messenger (mRNA) relative level of *GR* was observed (*P* < 0.01). On the contrary, a significant reduction was found in the relative mRNA expression of *GT* and *GP* (*P* < 0.05). There were no significant differences in *GC* or *GS* expression.

## Discussion

Cryptocaryoniasis is currently difficult to control and causes significant issues for marine aquaculture. Although some chemicals such as copper sulfate and formalin [24, 25] show good efficacy in killing *C. irritans*, there are increasing concerns about environmental pollution and human and animal safety issues from high concentrations of these chemicals. Studies on the mechanism of resistance to chemicals could contribute to reducing chemical use through increased susceptibility [26]. In this study, the mechanism of *C. irritans* resistance to oxidative stress induced by zinc ions was investigated by untargeted metabolomic analysis. This technology is often applied to study the physiological response to toxic metal exposure [12]. For instance, metabolomics has been used to explore the effect of Cu on planktonic and microalgae [27, 28]. In this study, glutathione metabolism was found to be involved in the defense to oxidative stress induced by zinc ions. A similar study by Ramakrishna reported that glutathione was generated in radish to resist oxidative damage induced by zinc ions [29]. Soga et al. also reported that glutathione may be a new biomarker for oxidative stress in mouse liver [11]. Similarly, an increase in glutathione content in *C. irritans* was determined by metabolomics in this study. This indicates that glutathione in *C. irritans* could be a biomarker for oxidative stress induced by zinc ions.

Glutathione metabolism involves various enzymes which contribute to the synthesis and metabolism of glutathione. The content of glutathione or ratio of reduced glutathione and oxidized glutathione in organisms is dependent on these enzymes, which include GS, GR, GT and GPx [8]. In this study, at 6 h post-treatment with zinc ions, the mRNA level of *GS* and *GT* was increased, which may indicate that the progress of synthesis and metabolism in glutathione was accelerated. Furthermore, the mRNA level of *GR* was significantly increased, but *GT* or *GPx* was decreased at 24 h post-zinc ion treatment. This promoted the synthesis of reduced glutathione, which eliminated ROS to retain cellar redox homeostasis. However, there were no significant changes in the mRNA level of these enzymes. That may be because of the disorder in the life-cycle caused by ROS damage induced by excess zinc ions.

The ascorbate–glutathione pathway, also known as the Asada–Halliwell pathway, plays a critical role in detoxifying ROS [30]. This pathway consists of ascorbate, glutathione, and four enzymes, namely ascorbate peroxidase, monodehydroascorbate reductase, dehydroascorbate reductase, and GR, which can contribute to the effective scavenging of ROS. In this study, the ascorbate and glutathione content were significantly increased, which may be attributed to the increase in mRNA *GR* expression. This may be why the addition of ascorbate increased the excystment rate of protomonts after zinc ion treatment. The antioxidant efficacy of ascorbate has been confirmed in rats [31]. These results illustrate that the ascorbate–glutathione pathway in *C. irritans* is involved in the defense against oxidative stress induced by zinc ions.

Methionine is an aliphatic, sulfur-containing, essential amino acid that shows good efficacy in defending against oxidative stress by activation of endogenous antioxidant enzymes, including methionine sulfoxide reductase A/B and the biosynthesis of glutathione to counteract oxidative stress [32]. In this biosynthesis process, methionine is a critical source of cysteine to synthesize glutathione through methionine adenosyltransferase, S-adenosylhomocysteine hydrolase, cystathionine β-synthase, cystathionine γ-lyase, GS, and so on [32]. In this study, cysteine and methionine metabolism in *C. irritans* was related to zinc ion stress. Furthermore, the excystment rate of *C. irritans* increased significantly after l-methionine was added following treatment with zinc ions. This indicates that l-methionine could be involved in the defense against oxidative stress in *C. irritans* by promoting glutathione biosynthesis. A similar result was reported by Wang et al. [33], who highlighted that l-methionine induced an endogenous antioxidant response to suppress ROS-derived oxidative stress. However, another study by Patra et al. found no significant effect of methionine in defending against oxidative stress induced by lead [31]. Further studies are needed to confirm the efficacy of methionine in the defense against oxidative stress in *C. irritans*.

In summary, metabolomics analysis was used to investigate the resistance mechanism of *C. irritans* to oxidative stress induced by zinc ions. Glutathione metabolism is involved in the antioxidant defense system in *C. irritans* to resist zinc ion stress, which could be an effective target for controlling cryptocaryoniasis.

## Supplementary Information

**Additional file 1: Fig. S1. **Multivariate analysis to *Cryptocaryon irritans* samples on Zn-treatment and control group. PCA: principal component analysis; PLS-DA: partial least squares-discriminant analysis; OPLS-DA: orthogonal projections to latent structures discriminant analysis

**Additional file 2: Fig. S2. **Changes in metabolites belonged to amino acids, peptides and analogues. Bars represent the mean (n=6)

**Additional file 3: Fig. S3. **Changes in metabolites belonged to carbohydrates and carbohydrate conjugates. Bars represent the mean (n=6)

**Additional file 4: Fig. S4. **Changes in metabolites belonged to lipids and lipid-like molecules. Bars represent the mean (n=6)

**Additional file 5: Fig. S5. **Changes in metabolites belonged to nucleosides, nucleotides and analogues. Bars represent the mean (n=6)

**Additional file 6: Fig. S6.** Amino acid metabolism pathways in *Cryptocaryon irritans* respond to zinc ions stress

**Additional file 7: Fig. S7.** Biosynthesis of other secondary metabolites pathways in *Cryptocaryon irritans* respond to zinc ions stress

**Additional file 8: Fig. S8.** Carbohydrate metabolism pathways in *Cryptocaryon irritans* respond to zinc ions stress

**Additional file 9: Fig. S9.** Xenobiotics biodegradation and metabolism pathways in *Cryptocaryon irritans* respond to zinc ions stress

**Additional file 10: Fig. S10.** Lipid metabolism pathways in *Cryptocaryon irritans* respond to zinc ions stress

**Additional file 11: Fig. S11.** Cofactors and vitamins metabolism pathways in *Cryptocaryon irritans* respond to zinc ions stress

**Additional file 12: Fig. S12.** Nucleotide metabolism pathways in *Cryptocaryon irritans* respond to zinc ions stress

**Additional file 13: Table S1.** Primers for RT-qPCR

## Data Availability

All datasets generated for this study are included in the manuscript/supplementary files.
