# Supplementary figures and images for: Glutathione metabolism in Cryptocaryon irritans involved in defense against oxidative stress induced by zinc ions
Source: Parasit Vectors. 2022 Sep 7;15:318. doi: 10.1186/s13071-022-05390-9 (PMC9454189; doi:10.1186/s13071-022-05390-9)

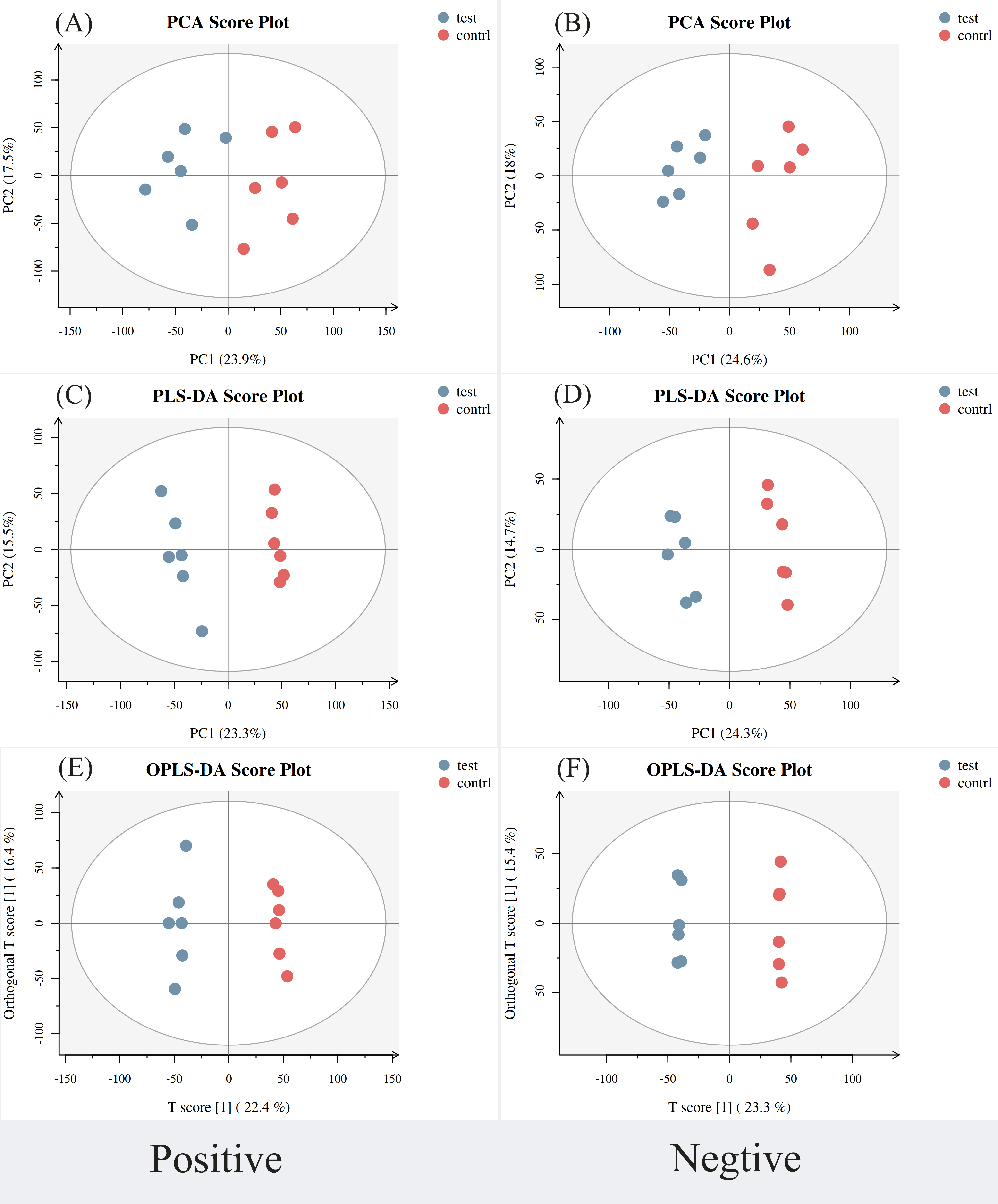

Supplement: Supplementary file 1 — Additional file 1: Fig. S1. Multivariate analysis to Cryptocaryon irritans samples on Zn-treatment and control group. PCA: principal component analysis; PLS-DA: partial least squares-discriminant analysis; OPLS-DA: orthogonal projections to latent structures discriminant analysis [file 13071_2022_5390_MOESM1_ESM.tif]
