# Supplementary material for: Glutathione metabolism in Cryptocaryon irritans involved in defense against oxidative stress induced by zinc ions
Source: Parasit Vectors. 2022 Sep 7;15:318. doi: 10.1186/s13071-022-05390-9 (PMC9454189; doi:10.1186/s13071-022-05390-9)
Supplement: Supplementary file 13 — Additional file 13: Table S1. Primers for RT-qPCR [file 13071_2022_5390_MOESM13_ESM.docx]

| Table S1 Primers for RT-qPCR | |
| --- | --- |
| Primer | Sequence (5′ to 3′) |
| *GC* F  *GC* R | TATGGATGCAATGGGATTCG  CCATCTGGTGTCAAAGTTTCC |
| *GR* F | GGAAGAAAGGCTGACACCAA |
| *GR* R | CAGCAAATATAGCCGGAGGA |
| *GT* F | TCTCCAAACCCAACAACCTC |
| *GT* R | CATCAAAGCTGAAGGAGAAGG |
| *GS* F | AGCCAGTGCCAAGATTAGGA |
| *GS* R | AGCCACTCCTCCTTCATTAG |
| *GPx* F | CGCCAAAGACATAGATGGAG |
| *GPx* R | AACCAGTGCGAACCATTTCT |
| *EF-1 β* F | GGAGATGATGATGATAATGATGA |
| *EF-1 β* R | CCAAACTAAACCTTCCAACT |
